# Notes and News

**Published:** 1890-03-29

**Authors:** 


### Notes and News.

Collected and Communicated.

Dr. Ainley points out the need of a new building to replace the present inconvenient Borough Hospital of Halifax. The patients in the hospital numbered more last year than ever before, and increased accommodation is an absolute necessity. A more cheerful site than the present suggestive one in a cemetery should be decided on, and the building should be commenced at once, so that the splendid new infirmary should not obtain an unfair start.

The Mayor of Birmingham has formulated his scheme for inquiring into hospital abuse. It is thought that no advantage will be gained by having a large committee, and the Mayor therefore suggests that four members representing hospitals (not being medical men), one member representing the Hospital Saturday Committee, and three representing the medical profession, together with the chairman, should constitute the committee, thus making nine members in all. The meetings will be held on Wednesday afternoons, and the press will be admitted to all except the first meeting.

An interesting meeting in aid of the Metropolitan Hospital was held at the Mansion House on Monday last, the Lord Mayor presiding, and Sir E. H. Currie and others being amongst the speakers. Since the Metropolitan Hospital dropt the title of "Free," and began work on provident principles its success has been curiously watched by those interested in the reform of the out-patient departments of our large hospitals. At the end of last year, *i.e.*, two years and two months after the starting of the provident department, there were 8,126 membership books issued, representing over 16,000 lives, and this proves that the poor really do appreciate this principle, by which a man is enabled to help himself, and not accept charity. In-patients were first admitted into the wards of the new hospital in January, 1888, the committee commencing by opening two small wards for accidents; since then, little by little, additional beds have been opened, so that at the end of 1889 76 beds were available for in-patients, viz., 24 for men, 24 for women, 16 cots for children, and two small wards devoted entirely to the members of the Jewish faith, to hold 12 beds. The Earl of Derby has consented to preside at the festival dinner on May 6th, when it is hoped £6,000 will be subscribed to pay off the debt on the building account.

Professor Vivian B. Lewis delivered an excellent lecture on "Our Atmosphere in its Relation to Health" at Mile End Assembly Hall last Tuesday, and will deliver another at Bethnal Green Memorial Hall at 8 p.m. on Tuesday next. These lectures are free "People's Lectures," and do a great deal of good in teaching the elements of health to those who are only too fond of a foul atmosphere. The lectures are illustrated by experiments.

The annual statement of the Norfolk and Norwich Hospital, to be submitted at the annual meeting on April 12th, is very full and very interesting. The hospital seems to be economically conducted; the average cost per occupied bed is £53 1s. 6d., the expenditure on wines and spirits last year was only £90, but for ale and porter it was £156. The number of in-patients last year was 1,354, and out-patients 4,302. The hospital needs an increase of annual subscribers, and, seeing that the Prince of Wales heads the list these ought to be forthcoming.

There is a sort of glamour over "St. Andrews by the Northern Sea" to every Scotchman, and even the report of the Cottage Hospital there is not like other reports. When the names of Shairp and Syme, and Adamson and Home Bell are connected with an institution, it is less to the figures than the letterpress we turn, and truly this year we are well rewarded for so doing. Mrs. Shairp, to arouse interest in the little hospital she serves so well, has published some verses by the late Principal Shairp written on the death of Sir James Simpson in 1870. We hope to quote freely from these verses on another occasion; for the present we can but say that we wish the Cottage Hospital every success.

The Governors of St. Thomas's Hospital showed a due sense of their responsibilities at the election of a treasurer on Tuesday last. There were two candidates—Sir Henry Knight, and Mr. J. Gadesden Wainwright, the latter of whom was elected by 117 votes to 57. Mr. Wainwright has for some years devoted a large amount of time, as an almoner, to the administrative work of St. Thomas's Hospital. He is a gentleman of strong character, excellent principles, and sound sense, all of which were pleasantly exhibited in his speech to the governors after his election. We regard the appointment of Mr. Wainwright as marking a new departure for the better in the government of the royal hospitals, and believe that Mr. Wainwright's assumption of the office of treasurer will be fruitful in beneficial results to the noble institution which has been entrusted to his management and control.

The sixteenth annual meeting of the Dublin Hospital Sunday Fund was held on March 12th, the Provost of Trinity College presiding. Dr. Grimshaw read the report of the council, which stated that the total amount of contributions for the year 1889 was £4,155 5s. 4d., being an increase of £25 15s. 1d. as compared with the previous year. The Distribution Committee last year were directed to inquire as to the expenditure per bed maintained by each of the participating hospitals, and the proportions which, in each case, establishment charges bear to maintenance, and to consider and to report to the council as to the desirability of modifying the distribution scheme, so that economy of hospital administration may be an important element in the assessment of the amount of the grant to each hospital. The committee, after investigation, confess that they are unable to solve these difficulties, and thus arrive at any equitable conclusions such as they could suggest that the council should embody in amendment of the distribution rules. The council are of opinion that a large amount of latitude should be permitted to hospital managers, especially as it appears from the reports of the Visiting Committee that, as a rule, the various managers succeed in carrying out the objects of their trusts in a creditable manner, though working in different ways, and apportioning their expenditure in different manners.

The following vacancies are announced this week, the amount of the salary in each case being given after the name of the institution:

**Resident Medical Officers.**

Bethlem Hospital, Two Clinical Assistants—Board, etc.
Leeds Infirmary, Surgical Officer.
Cardiff Infirmary, House Surgeon—Board, etc.
Hospital for Consumption, House Physician.
Hospital for Diseases of the Throat, Medical Officer—£50, board, etc.
Radcliffe Infirmary, House Physician—£80, board, etc.
Wandsworth Infirmary, Junior Assistant Medical Officer.
Metropolitan Hospital, House Physician—£60.
Metropolitan Hospital, House Surgeon—£60.
South-Western Fever Hospital, Clinical Assistant—Board, etc.
Oxford Eye Hospital, House Surgeon—£50, board, etc.
Ipswich Hospital, Assistant House Surgeon—£20, board, etc.

**Non-Resident Medical Officers.**

Chelsea Hospital for Women, Dispenser—£70.

City of London Hospital for Diseases of the Chest, Pathologist—£100.

Gloucester Infirmary, Assistant Physician.

Leicester Infirmary, Honorary Dental Surgeon.

Edinburgh Royal College of Physicians, Superintendent of the Research Laboratory—£200.

Royal Westminster Ophthalmic Hospital, Surgeon and Assistant Surgeon.

West London Hospital, Surgeon Dentist.

King's College, London, Professor of Psychological Medicine.

University of Edinburgh, Additional Examiner in Medical Jurisprudence—£90.

Metropolitan Hospital, Obstetric Physician.

---

The report of the Charity Organisation Society on hospital accounts, with which we cannot deal fully to-day, is remarkable in more ways than one. It proves to demonstration that gentlemen who have no technical knowledge of the special requirements of particular fields of work should refrain from expressing opinions thereon, and especially from formulating definite proposals and forms which reveal an ignorance of technique as amusing as it is instructive. The Charity Organisation Society has done much excellent work in many ways, but its latest report is neither calculated to increase the reputation of those responsible, nor to win public confidence for its proposals. The truth is, the report leaves matters, so far as the accounts of hospitals are concerned, exactly where it found them, and for practical purposes it might just as well have been published twenty years ago as to-day, the forms of account being mainly out of date.

---

Market Drayton has determined to have a Cottage Hospital, and the proposal to establish one at that place met with unanimous acceptance at a public meeting held at the beginning of this month at the County Court Room in that town. So anxious were those who attended the meeting to provide what has been for some time felt to be a necessity, that a large proportion of the amount required for building purposes was promised before the meeting separated, besides some £64 in annual subscriptions. Lady Mary Herbert, who has greatly interested herself in this matter, about seven years ago put forward a scheme for the provision of some such accommodation for sick persons. This scheme was then divided into three parts, and it was proposed to carry out such portions as it was found the funds would allow. The three portions were: (1) Obtaining resident trained nurses for the poor; (2) fitting up a small house with beds for cases of accident; and (3) providing a Provident Dispensary. The subject was at the time thoroughly discussed, with the result that only the first and last parts of the scheme were adopted, on such lines as have been worked on ever since. The Dispensary has been found to be most successful, but it has been felt that this Dispensary is available practically only to those persons resident in the town. In order, therefore, to extend the work, it has been thought that a Cottage Hospital is necessary, and on these grounds the proposal now adopted has been brought forward.

In connection with University College Hospital is an invalids' dinner table, at which semi-convalescents are given a good dinner of meat, bread, vegetables, and porter on the payment of twopence. There is also a children's table twice a-week, at which weak or puny children get a good dinner for a penny. If anyone wants to see how this new plan acts, they had better go to 2, Woburn Buildings, Euston Road, any day at twelve; when they have seen, marked, and learned, they had better subscribe to this charity, and consider carefully whether the scheme is not worthy of imitation in other neighbourhoods.

---

The treatment and cure of insanity is the great subject in medical circles just now, but is not the subject of the treatment and cure of inanity equally worthy of consideration? What the world suffers at the hands of the inane who roam unhindered in its midst, is treble what it suffers from the insane. The man who is actually mad is not allowed to publish novels or to play the violin in public; he is not permitted to air his vapid speeches before a scientific audience; he is refused admittance at St. Stephen's. But the inane man takes his inanity where he chooses, and inflicts on nervous and irritable humanity an amount of suffering he is utterly incapable of feeling himself. The physician who really wishes to benefit the bulk of mankind will take up the study of the cure of inanity.

---

Wednesday was a very busy day in the hospital world. To begin with, Mr. Gladstone went down to Guy's to open the new medical college, and received a very hearty welcome from the large concourse of visitors assembled. Even the counter-attraction of the boat-race did not seem to have enticed away a single student, and the proceedings went with *éclat*. The foundation-stone of the new wing of the Children's Hospital in Great Ormond Street was laid in the presence of a few old friends of the hospital, and as usual all visitors received a hearty welcome to this most hospitable of all our institutions. The walls of the new wing are already several feet high, so that the ceremony was somewhat of a farce. At the Hospital for Epilepsy, Regent's Park, e Bishop of Marlborough presided at the annual meeting, and a satisfactory report was adopted.

---

Two reports reach us from Birmingham, the one eminently unsatisfactory, the other fairly good. To get through disagreeable work first, is not that hospital an utter failure which confesses to a death-rate of 10·3 per cent., and gives as the cause the sanitary defects of the building? This is the tale told of the Birmingham and Midland Hospital for Women, established in 1871, and which has Mr. Lawson Tait on its staff. Of the deaths recorded, eight occurred in two groups, five in the last fortnight in August, and three in the last week in October and the first week in November, some of the deaths in each case being from septic poisoning. The hospital was closed during the month of September, and thoroughly cleaned and whitewashed. A committee was also appointed, but their recommendations have not yet been carried into effect. Alas! for red-tapism! In a case like this even debt should be contracted rather than use an unhealthy ward for a day. Then Mr. Lawson Tait is fighting hard for a better class of nurses, but Mr. Joseph Chamberlain, with his great knowledge of the subject, says there is no reason why nurses should not scrub the floors. Is it not possible that a surgeon may know more on this subject than a politician? But let us turn to a more pleasant report. The General Hospital treated over 49,000 patients last year, at a total expenditure of £15,385. This hospital has not a very good site, and could they secure a better would profit by the late Miss Rylands' benevolent bequest. The committee have, however, scoured the town in vain for a site, both central and open. They hope for success in the future. The Duke of Westminster was elected president of the hospital.

